# Defining Autism: Variability in State Education Agency Definitions of and Evaluations for Autism Spectrum Disorders

**DOI:** 10.1155/2014/327271

**Published:** 2014-06-02

**Authors:** Malinda L. Pennington, Douglas Cullinan, Louise B. Southern

**Affiliations:** ^1^Special Education, Department of Curriculum, Instruction, and Counselor Education, NC State University, Poe Hall 602, Campus Box 7801, Raleigh, NC 27695-7801, USA; ^2^North Carolina State University, Raleigh, NC 27607, USA

## Abstract

In light of the steady rise in the prevalence of students with autism, this study examined the definition of autism published by state education agencies (SEAs), as well as SEA-indicated evaluation procedures for determining student qualification for autism. We compared components of each SEA definition to aspects of autism from two authoritative sources: Diagnostic and Statistical Manual of Mental Disorders (DSM-IV-TR) and Individuals with Disabilities Education Improvement Act (IDEA-2004). We also compared SEA-indicated evaluation procedures across SEAs to evaluation procedures noted in IDEA-2004. Results indicated that many more SEA definitions incorporate IDEA-2004 features than DSM-IV-TR features. However, despite similar foundations, SEA definitions of autism displayed considerable variability. Evaluation procedures were found to vary even more across SEAs. Moreover, within any particular SEA there often was little concordance between the definition (what autism is) and evaluation procedures (how autism is recognized). Recommendations for state and federal policy changes are discussed.

## 1. Introduction

Autism spectrum disorder (ASD) refers to a group of pervasive neurodevelopmental disorders that involve moderately to severely disrupted functioning in regard to social skills and socialization, expressive and receptive communication, and repetitive or stereotyped behaviors and interests [1]. Only two decades ago ASD was considered rare, occurring or detected in about 1 in 1,000 children [2, 3]. Current estimates place the prevalence of ASD at 1 in 88 children [4], which suggests that roughly 800,000 US persons under age 20 have ASD.

In 1990, the US Congress amended the federal special education law (now called Individuals with Disabilities Education Improvement Act, or IDEA-2004) to make* autism* a category of education disability. Autism is defined for federal special education administrative purposes as stated below.

- Autism means a developmental disability significantly affecting verbal and nonverbal communication and social interaction, generally evident before the age of three that adversely affects a child's educational performance. Other characteristics often associated with autism are engagement in repetitive activities and stereotyped movements, resistance to environmental change or change in daily routines, and unusual responses to sensory experiences.
- Autism does not apply if a child's educational performance is adversely affected primarily because the child has an emotional disturbance, as defined in paragraph (c) (4) of this section.
- A child who manifests the characteristics of autism after the age of three could be identified as having autism if the criteria in paragraph (c) (1) (i) of this section are satisfied [5].

Autism is increasing in schools as well as in society. The US Department of Education (U.S. ED), which must monitor prevalence of autism and other educational disabilities, reported that US students with autism numbered 15,580 in 1992 [6]; by 2011 they numbered 406,957 [7].

The federal definition of autism preceded the fourth edition of the Diagnostic and Statistical Manual (DSM-IV) [8], and it is essentially unchanged since 1990. The federal definition is generally compatible with both the category of Pervasive Developmental Disorder (PDD) as described in DSM-IV and Autism Spectrum Disorder as described in DSM-5 [9], but it does not match any specific variety of PDD (see below). Within public school systems, students who have been clinically diagnosed with either a DSM-IV PDD or with DSM-5 Autism Spectrum Disorder are likely to be identified under the federal category of autism for the purpose of receiving special education services. Herein we use the term autism to include diagnoses of a DSM-IV PDD or DSM-5 Autism Spectrum Disorder.

In contrast to the IDEA-2004 definition, criteria for autism are more specific in the DSM-IV clinical diagnostic criteria. The* text revision* version [8] described four subcategories of PDD, including autistic disorder, Asperger's disorder, and a* not otherwise specified* subcategory (PDD-NOS) for presentations that clearly evidence autism but do not meet criteria for any named subcategory. In order to have qualified for DSM-IV autistic disorder, for example, a child must have exhibited a total of at least six listed characteristics, including at least two characteristics of “qualitative impairment in social interaction,” one characteristic of “qualitative impairments in communication,” and one characteristic of “restricted, repetitive and stereotyped patterns of behavior, interests, and activities” [8]. Additionally, DSM-IV autistic disorder has criteria that address age-of-onset and ruling out other conditions.

Recently, DSM-IV was superseded by DSM-5 [9]. The disorders comprising PDD in DSM-IV are largely addressed in DSM-5 by the Autism Spectrum Disorders category, which—unlike DSM-IV's PDD—has no subcategories. However, identification criteria still include substantial social problems (social initiations and responses, nonverbal social communication, and social relationships) and restricted, repetitive behaviors or interests (deviant speech or movements, rituals and resistance to change, preoccupations, and sensory reactivity). State education agencies (SEAs) have not yet incorporated DSM-5 information into their policies, procedures, and practices related to students with autism, and the DSM-5 definition was not involved in the present study. Implications of DSM-5 criteria for autism are presented in Section 4.

States also have administrative definitions of various categories of education disability, including autism. State education agency (SEA) definitions of a disability do not have to match the federal definition but must substantially address its elements or lose federal financial support for special education. Thus it is puzzling that the percentage of prevalence of autism varies greatly across different states. In 2012, for instance, when the mean rate of autism for all SEAs was 0.6% of the student-age population, Iowa reported the lowest prevalence at 0.1%, while Minnesota reported the highest rate at 1.2% [7]. No doubt the prevalence of ASD naturally varies somewhat with geography [4] but probably not by such a large factor, greater than tenfold in adjacent states. Conceivably, some state-by-state variation might be attributable to the content of SEA definitions of autism and perhaps the evaluation procedures required to accurately measure the concepts presented in definitions.

In a study of SEA definitions of autism, MacFarlane and Kanaya [10] found substantial variation in the eligibility criteria used by different states. By their analysis, 35% of SEAs based autism eligibility solely on the federal definition of autism, while 65% used diverse other criteria including symptoms of autism from the DSM-IV-TR. However, these researchers encountered difficulty obtaining eligibility information directly from SEAs through their websites and email requests to SEA administrators, so they based their findings on a review of special education definitions contained in state legal code documents (repositories of laws and supportive regulations). MacFarlane and Kanaya [10] noted that one limitation of this process is that state legal terminology may not accurately reflect SEA policies and practices.

MacFarlane and Kanaya [10] also considered SEA evaluation procedures for autism eligibility: they recorded the presence or absence of a requirement for clinical diagnosis of autism by a pediatrician or qualified clinician. However, this was the limit of their analysis of SEA evaluation procedures and their link to autism eligibility criteria. Because eligibility evaluation procedures can help operationalize a definition of autism, there clearly is a need to explore SEA evaluation information to a greater extent.

Federal special education law addresses, in a general way, evaluation procedures for determining whether a student is diagnosed as having a disability, including autism: “The child is assessed in all areas related to the suspected disability, including, if appropriate, health, vision, hearing, social and emotional status, general intelligence, academic performance, communicative status, and motor abilities” [11]. Based on this guidance, each state determines evaluation procedures, and these may be further modified by individual school districts. Obviously, this could lead to a wide variety of evaluation procedures implemented by diverse evaluators. It would be good to know what evaluation procedures different SEAs use for autism eligibility; however, we are aware of no compilation of them. To accurately determine eligibility, evaluation procedures should appropriately measure criteria established by SEAs. Similar criteria across states might be expected to call for similar evaluations. If evaluation procedures do not align with stated criteria or vary greatly across states, they throw into question the accuracy of autism identification by SEAs.

The purpose of the present study was to consider the current status of SEA definitions of autism and SEA evaluation procedures for autism eligibility. We wanted to analyze components found in the definitions in order to compare definitions to their probable sources and to each other. We also wished to analyze the evaluation procedures in order to compare them across SEAs and to consider the extent to which SEA evaluation procedures address concepts contained in the SEA definitions of autism.

## 2. Method

### 2.1. Data Collection

We looked for SEA definitions of autism for each of the states and the District of Columbia by accessing SEA websites and searching for “special education services” and as necessary, additional terms such as “rules and regulations” and “autism.” Some SEAs had more than one apparently official definition of autism, presented on multiple SEA websites. In this instance, we coded the definition which included reference to state legal code. Some SEAs presented not only a statewide definition but additional definitions apparently reserved for particular school districts in the state. For this occurrence, we coded just the definition for the entire state.

For many SEAs, evaluation procedures for autism were located within the same website that stated the definition. If not, we searched the SEA website using “autism eligibility.” All SEA definitions of and evaluation procedures for autism were collected between February and May, 2012.

### 2.2. Coding SEA Definition Components

To analyze and compare SEA definitions, we developed a list of ASD* components* based on consideration of three sources: (a) the definition of autism found in IDEA-2004, (b) the description of Autistic Disorder in DSM-IV-TR [8], and (c) the description of Childhood Autism in the tenth edition of* International statistical classification of diseases and related health problems* [12]. Our review of the descriptions of Asperger's Disorder and PDD-NOS in DSM-IV-TR showed that the criteria for these subcategories of PDD were subsumed by the criteria for Autistic Disorder (characteristics were the same, although not necessarily to the same degree). Therefore, our final list of definition components relied heavily on the main features of Autistic Disorder in DSM-IV-TR. We supplemented these with two components from the IDEA-2004 definition of autism that do not overlap with the DSM-IV-TR material: sensory processing problems and emotional disturbance exclusion (i.e., IDEA-2004 states that if the student qualifies for special education under the emotional disturbance disability, he or she cannot qualify under the autism disability). We judged that the characteristics of ICD-10 Childhood Autism are essentially similar to DSM-IV-TR Autistic Disorder, so the ICD-10 contributed no unique components to our list.

Our initial reading of the SEA definitions confirmed the suggestion of MacFarlane and Kanaya [10] that some SEAs appeared to rely heavily on either the DSM-IV-TR criteria or the IDEA-2004 statement for their definitions of autism. Therefore, we added two more components to the coding list:* Essentially DSM* and* Essentially IDEA*. We marked a SEA definition as Essentially DSM if it presented the three DSM-IV-TR set A criteria for autistic disorder (briefly stated as impaired social functioning, impaired communication, and stereotyped interests and activities), including the four specific subcriteria for each criterion, with no more than minor wording changes (e.g., “significant impairment” instead of “qualitative impairment”). We also coded Essentially DSM even if the DSM-IV-TR criterion B (onset before age 3) or criterion C (exclusion of Rett's disorder and childhood disintegrative disorder) was not included in the SEA definition. On the other hand, we marked an SEA definition as Essentially IDEA if it embodied either the entire IDEA-2004 definition or the major phrases in it verbatim.

### 2.3. Coding Evaluation Features

We developed a second coding list to analyze and compare SEA diagnostic evaluation procedures for autism. We began with the evaluation statement in IDEA-2004 regulations (which covers any educational disability, not just autism): “The child is assessed in all areas related to the suspected disability, including, if appropriate, health, vision, hearing, social and emotional functioning, general intelligence, academic performance, communicative status and motor abilities [11].”

Our evaluation coding list included each of the above statement's evaluation areas as* features*. In addition, our initial reading of the SEA evaluation statements revealed some other evaluation areas that we added to the list of features. These additions included the following: medical evaluation or information, observation, parent interview, social-developmental history, behavioral scale, adaptive behavior, sensory functioning, autism-specific diagnostic tool, and developmental assessment.

Following practice with coding, the authors clarified coding procedures through discussion. For example, SEA evaluation statements that included* psychological* assessment were coded as individual intelligence assessment and, if the statement indicated, additional psychological procedures. The first author coded the definition component list and the evaluation features list for all SEA definitions and all SEA evaluation statements. The second and third authors each coded definition components and evaluation features for an assigned half of the SEAs.

### 2.4. Coding Agreement

Intercoder agreement was calculated separately on each definition component and evaluation feature by dividing the number of coding agreements by the number of agreements plus disagreements. Disagreements between coders were resolved through discussion and review of material; if disagreements remained, the first author's coding was used.

## 3. Results

We obtained current SEA definitions of autism, as well as eligibility evaluation procedures for that category of special education for all states and the District of Columbia, from each SEA's website. We coded this information according to the definition and evaluation lists we created. Initial agreement (prior to discussion to achieve consensus) per coding item ranged from 0.88 to 1.0 with a mode of 0.96.

### 3.1. Basis of Definitions

A strong majority of SEA definitions of autism (*n* = 35, 69%) were coded as Essentially IDEA (IDEA-2004 definition entirely or in major part) as the sole basis of autism eligibility criteria for their state. Only 4 SEA definitions (8%) were considered Essentially DSM (all three DSM-IV-TR criteria for Autistic Disorder (impaired social functioning, impaired communication, and stereotyped interests and activities), including the four specific subcriteria for each). Additionally, 10 SEA definitions (20%) used both the IDEA-2004 and the DSM-IV-TR criteria, incorporating essential elements from both sources either verbatim or with no more than minor wording changes. Two SEAs, California and Florida, presented unique state definitions that addressed some characteristics of autism such as those found in IDEA-2004 and DSM-IV-TR but did not contain enough similarity to be coded as either Essentially IDEA or Essentially DSM.

After definition basis was determined, we calculated a point-biserial correlation to investigate whether a relationship existed between source of definition (IDEA or DSM) and autism prevalence reported by states to the US Department of Education [7]. The analysis revealed no relationship based on this factor (*r*_37_ = −0.11, *P* > .05).

### 3.2. Autism Eligibility Criteria

The number of components in SEA autism definitions ranged from 5 to 17. The modal number of components was 8. Results of our examination of various components in each SEA definition are summarized in Table 1.

*Social Interaction Impairment.* Regardless of what source they appeared to be based on, 46 (90%) of the SEA definitions stated that impaired social interaction is required for the student to be qualified under the autism education disability. As Table 1 shows, considerable numbers of definitions included particular forms of social interaction impairment (impaired nonverbal social, lack of peer relationships, lack of spontaneous joint attention, lack of social-emotional reciprocity) as criteria for autism. In some definitions the particular forms were additions to a general statement about social interaction impairment, while in other definitions there was no general statement, just one or more of the particular forms of social interaction impairment.

*Communication Impairment.* Table 1 also shows that 46 (90%) of the SEA definitions contained a general statement to the effect that impairment in communication is a necessary characteristic to qualify for autism. Quite a few state definitions also presented particular forms of communication impairment as criteria for autism (impaired spoken language, impaired conversation ability, stereotyped language, impaired fantasy or social play). Again, in some but not all SEA definitions the particular forms were additions to a general statement.

*Restricted, Repetitive, and Stereotyped Behaviors and Interests.* Only 2 SEA definitions presented a general statement that to qualify for autism the student must show restricted or stereotyped behaviors or interests. However, all the definitions stated that one or more of the particular forms of restricted, repetitive, or stereotyped behaviors and interests had to be present for the student to qualify. Specifically, nearly all SEA definitions listed preoccupation with stereotyped interests, nonfunctional routines and rituals, and stereotyped mannerisms, while about one-third of definitions listed preoccupation with object parts.

*Other Components.* Among the other key features, 46 definitions (90%) stated that onset prior to three years of age was either generally evident or required. Of these, 41 stated that exceptions to the age of three onset guideline or requirement are permitted; 5 definitions did not state an age of onset.

An eligibility statement about unusual responses to sensory experiences was present in 44 SEA definitions (86%). The definitions contained six different exclusion conditions (see Table 1), most commonly identification under the emotional disturbance category of special education (86%). On the other hand, three states specified conditions that could coexist with autism eligibility.

### 3.3. Autism Evaluation Features

The number of SEA autism evaluation features ranged from 0 to 15 with 8 as the modal number of criteria. Only 10 SEAs used all, and only, features of the IDEA evaluation statement to indicate their evaluation criteria for autism eligibility. However, as Table 2 shows, the majority of SEAs used one or more features of the IDEA evaluation statement. Specifically, health evaluation was noted in 57% of SEA positions; vision evaluation in 57%; hearing evaluation in 57%; social and emotional evaluation in 59%; academic evaluation in 88%; speech-language evaluation in 88%; motor skills evaluation in 88%. In addition, the following other evaluation features were present in the majority of SEA evaluation statements: observation, 51%; parent interview, 53%; social-developmental history, 53%; psychological, 84%; adaptive behavior, 51%. Table 2 presents additional features that we found in less than 50 percent of SEA evaluation procedures. Among these less-recommended features is the requirement to administer an autism-specific evaluation as part of the eligibility process. Of the 15 SEAs (29%) that included an autism assessment in the evaluation process, none specified the use of a recognized instrument such as the Autism Diagnostic Observation Schedule (ADOS) [13] or the Childhood Autism Rating Scales (CARS) [14]. Although 3 of these SEAs did indicate the required use of a state-created autism checklist, none gave any reference to a source or psychometric characteristics of those checklists.

## 4. Discussion

The present study revealed that definitions of autism and the eligibility evaluation procedures for it are readily available on SEA websites. Most SEA definitions substantially resemble the federal definition of autism found in IDEA-2004, including its components about deviant responses to sensory experiences and exclusion of students with emotional disturbance. Many definitions have in addition some elements of DSM-IV-TR autistic disorder. On the other hand, only one-fifth of SEA evaluation procedures substantially reflect the IDEA-2004 statement on assessment for disability eligibility, although many SEAs incorporate a few aspects of that statement.

Study results suggest several points that call for criticism, but first we acknowledge that it is far easier to disapprove of existing definitions and evaluation statements about autism than to present demonstrably superior alternatives. That said, below we point to several drawbacks to the existing SEA information and offer a direction that could lead to their improvement.

Table 1 shows that most SEA definitions prohibit qualification under the category of autism for a student who has certain other conditions. This is a flaw because some of those other conditions are commonly found among children with autism. For example, children with autism often experience mood disorder, anxiety disorder, and other mental disorders [15–17] that might qualify the student for emotional disturbance. Yet in 44 SEA definitions, qualifying for emotional disturbance excludes qualifying for autism.

Moreover, despite the frequent definition emphasis on ruling out an emotional disturbance, only 11 SEAs (22%) require a behavioral evaluation that would document the presence or absence of emotional-behavioral problem characteristics. Given this disconnect, it is possible that students with characteristics of autism may either not be so identified due to rule-out criteria or may be identified under the autism category but not receive proper services for their unevaluated mental health issues.

The DSM-5 definition of autism spectrum disorder recognizes the comorbidity of autism with other mental disorders, such as anxiety and obsessive compulsive disorder, and allows for specification of these coexisting conditions within the clinical diagnosis. SEAs that undertake a revision of their autism special education category may wish to follow the lead of DSM-5 in this regard by recognizing that students whose emotional and behavior problems might qualify them for emotional disturbance may more appropriately be qualified for autism.

In addition to coexisting mental health conditions, many cases of ASD are characterized by abnormal brain development [18, 19] with documented seizures present in perhaps as much as 30% of this population [16]. Our study revealed that 29 SEAs called for health evaluations and 18 called for medical evaluations; however, what these evaluations entail was left unstated or vague. Although 3 SEAs called for a neurological examination as part of the health or medical evaluation, we recognize that such a universal requirement would represent an enormous cost to school districts. However, we do recommend that SEAs clarify the content and purpose of stated health and medical evaluations including whether they represent the same information. If SEAs were to incorporate elements of the DSM-5 autism criteria, they would see that recognition of coexisting medical or genetic conditions is part of the ASD in DSM-5. Developing a comprehensive description of the student beyond the basic categories of social functioning, communication skills, and repetitive behaviors will provide schools and families with a more complete picture of student needs and the services required to meet them by a variety of providers.

Of course, substantial communication impairments do characterize a large fraction of students with autism. These include failure to speak, echolalia, and other severe problems of receptive and expressive language [20]. While 45 SEAs called for speech and language evaluation to qualify for autism, none specified and few suggested language problem areas to evaluate. Only 6 SEAs prescribed evaluation of the student's needs regarding augmentative or assistive technology for language. SEA evaluation procedures for autism may be improved by the addition of more specifics on language evaluation.

Similarly, the present study noted that the majority of SEAs (*n* = 44, 86%) consider the presence of unusual responses to sensory stimuli to be a core feature of autism. However, as with other noted discrepancies, only 9 SEAs (18%) required an evaluation procedure to document the existence and nature of possible sensory functioning difficulties. The DSM-5 criteria for ASD recognize the pervasiveness and impact of sensory issues on students with autism and include this feature prominently.

The increasing prevalence of autism in recent years has been attributed, to some extent, to a broadening of the concept of autism [21, 22] to include children whose social problems, communication problems, stereotyped behaviors and interests, and other problems are less severe than most cases diagnosed two or three decades ago. Similarly, present results suggest that the large increase in students qualifying for special education under autism may have been caused, in part, by vague definitions together with ambiguous, variable, and irrelevant evaluation procedures. A particular example would be the underutilization of recognized autism diagnostic instruments. Improvements in SEA definitions of autism will be of limited use unless they are accompanied by better assessment procedures that will support better evaluation and other aspects of assessment (e.g., measurement of student progress per characteristic of autism).

Present results and their implications compel us to add a couple of qualitative judgments about most of the SEA definitions of and evaluation statements for autism. First, for many SEAs the definition and the evaluation procedures unfortunately did not correspond. Definition components often were not addressed by evaluation features, even in a cursory way. Second, many of the definitions and evaluation procedures as presently stated are too vague to be of much use. For example, one state's definition says that autism is a disorder “significantly affecting verbal communication,” but its autism evaluation statement calls for evaluators to “assess all areas of need including… communicative status” [23]—hardly a specification of needed procedures or measurable criteria for verbal communication, suited for use in determining eligibility. Of course, that so many of SEA definitions and evaluation statements closely resemble those of the IDEA-2004 implies that the federal items are too vague as well. The federal and SEA definitions and evaluation procedures for autism must be improved.

There is no time like the present, literally, for making those improvements. As noted earlier, the number of students with autism continues to rise. This not only makes inferior definition and evaluation a disservice to more students but it also increases the number of parents and teachers who will probably support improved ones. With the publication of DSM-5, SEAs have the opportunity to expand and update their current definition of autism. The DSM-5 criteria encompass all of the elements stated by the current IDEA definition with the exception of allowing for the coexistence of potential emotional disorders rather than exempting autism eligibility based on those characteristics. Moreover, IDEA-2004 is overdue for Congressional reconsideration and possible amendment, so an opportunity window will open, but for a limited time, to also update and clarify the federal educational definition of autism.

Third, the DSM-5 concept of autism, like DSM-IV, emphasizes problems of social functioning and repetitive behaviors and interests, along with problems with social aspects of language. DSM-5 recognizes the salience of sensory processing problems and the possibility of coexisting mental health disorders. Officials of more SEAs should consider elements of DSM-5 autism spectrum disorders as they consider revisions to their state definition of autism and corresponding procedures by which assessors will provide data for eligibility determination.

The need is evident and the moment is favorable to make improvements to administrative definitions of and evaluation procedures for autism as an education disability on both the state and federal levels. Therefore, we propose that an appropriate national organization or agency seeks input and pursue consensus on this issue. Input should be obtained from representatives of special education and other professional groups, ASD advocacy organizations, state and federal education agencies, and other individuals and groups with significant interests in the appropriate education of students with autism.

An agenda might include addressing important questions, perhaps including some of the following: (a) what are the critical components that should be in an educational administrative definition of autism? (b) Are there other categories of educational disability that should be mutually exclusive with autism, and if so, how should this be presented? (c) Should the definition and evaluation procedures address the presence of autism overall, or its more specific components separately? (d) How is a balance established between evidence-based best assessment practices versus assessor professional judgment regarding individual student situations? (e) Are there critical validated or standardized evaluation procedures that should be specified, and if so, would SEA procedures that call for them incur large legal and financial obligations? As we see it, considering such fundamental questions will supplement other activities of participants to move toward the goal of this effort: to create ideal revisions to the IDEA definition of autism and its statement of evaluation procedures—in this case, procedures specific to autism eligibility.

If this goal is achieved, what might happen next? The model definition and evaluation procedures might be presented to federal authorities with the power to incorporate it into the next iteration of IDEA. They may be presented to SEAs for consideration in state-level revisions. Improved, more specific definitions and evaluation procedures will enable SEAs and school districts to better serve students with autism and better allocate resources. Ultimate outcomes such as school graduation, employment, and community involvement must begin with a correct first step. For autism, the first step is to accurately identify the disability and any accompanying individual conditions in a manner that students receive good services and opportunities regardless of where they live in our nation.

## Figures and Tables

**Table 1:** Components of state education agency autism definitions.

Definition component	Number of states	Percentage
Social interaction impairment—general statement	46	90
Impaired nonverbal social	15	29
Lack of peer relationships	21	41
Lack of spontaneous joint attention	14	27
Lack of social-emotional reciprocity	18	35
Communication impairment—general statement	46	90
Impaired development of spoken language	19	37
Impaired ability to initiate or sustain a conversation	17	33
Stereotyped and repetitive use of language	17	33
Lack of spontaneous make-believe or social imitative play	15	29
Restricted, repetitive, and stereotyped behaviors and interests—general statement	2	4
Preoccupation with stereotyped interests	49	96
Nonfunctional routines or rituals	50	98
Stereotyped and repetitive mannerisms	49	96
Preoccupation with parts of objects	17	33
Other components		
Onset prior to the age of three	47	92
Unusual sensory experiences	44	86
Exclusion conditions	45	88
Emotional disturbance	44	86
Intellectual/developmental delay	3	6
Pervasive developmental disorder	1	2
Rett's disorder or childhood disintegrative disorder	1	2
Schizophrenia	1	2
Visual or hearing impairments	1	2
Coexisting conditions allowed	3	6

**Table 2:** Features of state education agency evaluation procedures for autism.

IDEA evaluation features	Number of states	Percentage
Academic	45	88
Speech-language	45	88
Intelligence (or psychological)	43	84
Social and emotional	30	59
Health	29	57
Vision	29	57
Hearing	29	57
Motor skills	29	57

Other evaluation features		
Parent interview	27	53
Social-developmental history	27	53
Observation	26	51
Adaptive behavior	26	51
Medical evaluations or information	18	35
Autism-specific	15	29
Behavioral scale	11	22
Sensory functioning	9	18
Developmental assessment	5	10
